# A sulfate-reducing bacterial genus, *Desulfosediminicola* gen. nov., comprising two novel species cultivated from tidal-flat sediments

**DOI:** 10.1038/s41598-021-99469-5

**Published:** 2021-10-07

**Authors:** Jaeho Song, Juchan Hwang, Ilnam Kang, Jang-Cheon Cho

**Affiliations:** 1 Division of Microbiology, Honam National Institute of Biological Resources, 58762 Mokpo, Republic of Korea; 2 Department of Biological Sciences, Inha University, Incheon, 22212 Republic of Korea

**Keywords:** Microbiology, Systems biology

## Abstract

Tidal-flat sediments harbor a diverse array of sulfate-reducing bacteria. To isolate novel sulfate-reducing bacteria and determine their abundance, a tidal-flat sediment sample collected off Ganghwa Island (Korea) was investigated using cultivation-based and culture-independent approaches. Two Gram-stain-negative, strictly anaerobic, rod-shaped, sulfate-reducing bacteria, designated IMCC35004^T^ and IMCC35005^T^, were isolated from the sample. The two strains reduced sulfate, sulfite, elemental sulfur, thiosulfate, Fe(III) citrate, and Mn(IV) oxide by utilizing several carbon sources, including acetate. The 16S rRNA gene amplicon sequencing revealed that the tidal-flat sediment contained diverse members of the phylum *Desulfobacterota*, and the phylotypes related to IMCC35004^T^ and IMCC35005^T^ were < 1%. The two strains shared 97.6% similarity in 16S rRNA gene sequence and were closely related to *Desulfopila aestuarii* DSM 18488^T^ (96.1–96.5%). The average nucleotide identity, level of digital DNA–DNA hybridization, average amino acid identity, and percentages of conserved proteins determined analyzing the whole-genome sequences, as well as the chemotaxonomic data showed that the two strains belong to two novel species of a novel genus. Additionally, genes related to dissimilatory sulfate reduction were detected in the genomes of the two strains. Unlike the genera *Desulfopila* and *Desulfotalea*, IMCC35004^T^ and IMCC35005^T^ contained menaquinone-5 as the major respiratory quinone*.* Collectively, IMCC35004^T^ and IMCC35005^T^ were concluded to represent two novel species of a novel genus within the family *Desulfocapsaceae*, for which the names *Desulfosediminicola ganghwensis* gen. nov., sp. nov. (IMCC35004^T^ = KCTC 15826^T^ = NBRC 114003^T^) and *Desulfosediminicola flagellatus* sp. nov. (IMCC35005^T^ = KCTC 15827^T^ = NBRC 114004^T^) are proposed.

## Introduction

Sulfate-reducing bacteria (SRB) are defined by their ability to use sulfate as a terminal electron acceptor during anaerobic respiration, and they are known to be universally found in anoxic habitats, including marine sediments, soil, groundwater, active sludge, and animal guts^1–5^. SRB are particularly ubiquitous and abundant in anoxic marine sediments, accounting for up to 10–30% of the corresponding bacterial community^6–8^. Sulfate reduction, mediated by diverse groups of SRB, accounts for > 50% of the carbon re-mineralization in coastal sediments^9^, thereby constituting one of the most important metabolic processes accounting for the anaerobic degradation of the organic matter in anoxic marine sediments.

SRB and sulfate-reducing archaea are phylogenetically widespread, distributed among at least three phyla within the domain *Bacteria* (*Desulfobacterota*, *Nitrospirota*, and *Firmicutes)* and two classes of the domain *Archaea* (*Archaeoglobi* and *Thermoproteia*), respectively^10,11^. Of these, the phylum *Desulfobacterota*^11^ harbors > 80 genera of sulfate-reducing bacteria (https://lpsn.dsmz.de), representing the largest phylum harboring SRB. Among the many families of the phylum *Desulfobacterota,* the family *Desulfocapsaceae* was first proposed by Waite et al*.*^11^ via the reclassification of the family *Desulfobulbaceae* based on phylogenetic analyses with 120 conserved single-copy marker genes and 16S rRNA gene. As of August 2021, the family embraces five genera, *Desulfofustis*^12^, *Desulfocapsa*^13^, *Desulforhopalus*^14^, *Desulfotalea*^15^, and *Desulfopila*^16^, which have been identified from sewage sludge or marine, brackish, and freshwater sediments. Members of the family *Desulfocapsaceae* show various phenotypic characteristics, such as a wide temperature range for growth (− 1.8 to 40 °C), motile or non-motile property, anaerobic chemolithotrophic or chemoheterotrophic metabolism, anaerobic respiratory or fermentative growth, and usage of various electron donors and acceptors for sulfate reduction. According to the EzBioCloud database, which holds quality-controlled bacterial genomes^17^, members of the family *Desulfocapsaceae* have a 3.5–6.1 Mb genome with 45.4–56.3% G + C content. The family is considered one of the major sulfate-reducing bacterial groups in anoxic marine sediments, often comprising up to 11% of the total bacterial community in a habitat^8,18,19^.

The coastal area of the Yellow Sea is characterized by the presence of vast tidal-flat sediments, where the families *Desulfobulbaceae* and *Desulfocapsaceae* have been abundantly detected via culture-independent analyses, but the members of the family have rarely been isolated^20–22^. This study reports the isolation of two sulfate-reducing bacterial strains of the family *Desulfocapsaceae* from a tidal-flat sediment of the Yellow Sea. Based on the distinct genomic and phenotypic characteristics, the two strains were considered to belong to two novel species of a new genus in the family *Desulfocapsaceae*.

## Results and discussion

### Phylogenetic and phylogenomic analyses

**Figure 1:**
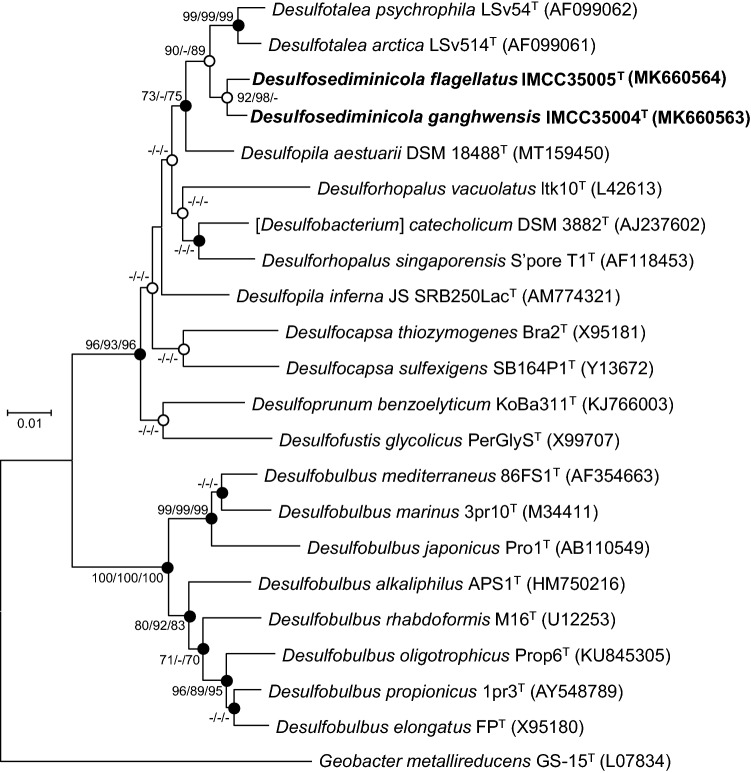
Positions of strains IMCC35004^T^ and IMCC35005^T^ on the neighbor-joining phylogenetic tree based on the 16S rRNA gene sequences. Filled circles indicate that the corresponding nodes were identified from all the treeing methods. Open circles indicate the nodes identified from the two treeing methods. The bootstrap values (expressed as percentages of 1000 replications) are shown at nodes for neighbor-joining, minimum-evolution, and maximum-likelihood methods; only the values ≥ 70% are shown. The GenBank/EMBL/DDBJ accession numbers are shown in parentheses. *Geobacter metallireducens* GS-15^T^ (L07834) was used as an outgroup. The bar represents 0.01 nucleotide changes per position.

**Figure 2:**
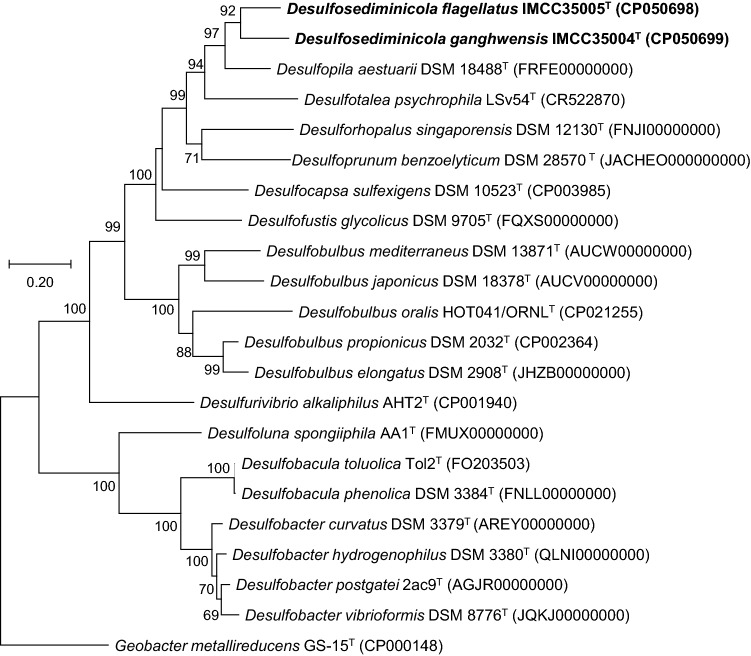
Positions of strains IMCC35004^T^ and IMCC35005^T^ on the phylogenetic tree based on the whole-genome sequences. A total of 29,457 amino acids were used for the phylogenomic analyses using up-to-date bacterial core gene sets (UBCG). The genome accession numbers are shown in parentheses. *Geobacter metallireducens* GS-15T (CP000148) was used as an outgroup. The bootstrap supports are shown at the nodes. The bar represents 0.2 substitutions per position.

Two sulfate-reducing bacterial strains, designated IMCC35004^T^ and IMCC35005^T^, were isolated from a sediment sample collected off the west coast of Korean Peninsula during a study on the culturable anaerobic bacterial community of the tidal flat in the eastern margin of the Yellow Sea. Comparative sequence analyses using the almost complete 16S rRNA gene sequences of strains IMCC35004^T^ and IMCC35005^T^ revealed that the two strains are affiliated with the family *Desulfocapsaceae*. The two strains were found to share 97.6% similarity in 16S rRNA gene sequence, which is less than the proposed cut-off value of 98.7% for bacterial species demarcation^23,24^, suggesting that these strains belong to two separate species. Strains IMCC35004^T^ and IMCC35005^T^ were identified to show the highest 16S rRNA gene sequence similarity with uncultured clone HS030 (JX391366; 99.7%) and strain LS5B (LR792819; 99.9%) retrieved from marine sediment. Among the validly published species, strains IMCC35004^T^ and IMCC35005^T^ were most closely related to *Desulfopila aestuarii* DSM 18488^T^ (96.5 and 96.1% 16S rRNA gene sequence similarity, respectively), followed by *Desulfotalea psychrophila* LSv54^T^ (95.7 and 95.9%) and *Desulfotalea arctica* LSv514^T^ (95.8 and 95.9%). Phylogenetic analysis based on the 16S rRNA gene sequences revealed that these two novel strains form a distinct and robust clade, with 92–98% bootstrap values, within the family *Desulfocapsaceae* (Fig. 1). The two strains formed a clade with the genus *Desulfotalea* (89–90% bootstrap supports) and a large clade with *Desulfopila aestuarii* DSM 18488^T^ (73–75% bootstrap supports). In contrast to the 16S RNA gene tree (Fig. 1), where strains IMCC35004^T^ and IMCC35005^T^ form a clade with the genus *Desulfotalea*, the phylogenomic tree based on the bacterial core-gene sets (Fig. 2) show that the two strains first cluster with *Desulfopila aestuarii* and form a clade with the genera *Desulfotalea* and *Desulforhopalus*. Thus, the two strains cannot be assigned to a specific genus.

The overall genome-relatedness indices supported that strains IMCC35004^T^ and IMCC35005^T^ belong to two separate species (Supplementary Table S1). The average nucleotide identity (ANI) and digital DNA–DNA hybridization (dDDH) values between the two strains were estimated at 70.2% and 20.5%, respectively, far below the ANI (95–96%) and dDDH (70%) thresholds proposed for bacterial species demarcation^25,26^. Accordingly, these two strains each represent a novel species. The values of average amino-acid identity (AAI) and percentage of conserved proteins (POCP) of the two strains and *Desulfopila aestuarii* DSM 18488^T^ were calculated to be 67.3–69.6% and 47.9–49.6%, respectively (Supplementary Table S1), suggesting that the two strains may belong to the genus *Desulfopila* or a new genus since the values are marginally higher or lower than the AAI (~ 65%) and POCP (50%) thresholds for genus delineation^27,28^. In contrast, the AAI and POCP values between the two strains and *Desulfotalea psychrophila* LSv54^T^ were calculated at 56.8–57.0% and 42.9–43.8%, respectively, indicating that the two strains belong to a genus different from *Desulfotalea*.

### Genomic characteristics

The genomes of strains IMCC35004^T^ and IMCC35005^T^ were estimated at 5,653,142 and 6,751,878 bp, respectively, and comprise single circular chromosomes, (Supplementary Table S2; and Supplementary Figs. S1 and S2). The G + C content of IMCC35004^T^ and IMCC35005^T^ genomes was calculated to be 48.9 and 44.3%, respectively (Table 1). IMCC35004^T^ and IMCC35005^T^ genomes were found to harbor 4715 and 5479 protein-coding, 73 and 76 tRNA, and 18 and 21 rRNA genes, respectively. Six and seven copies of 16S rRNA gene sequences were detected in IMCC35004^T^ and IMCC35005^T^ genomes, respectively. These copies showed 100% sequence similarity among themselves and with their Sanger-sequencing counterparts amplified using PCR.

**Table 1:** Characteristics that differentiate strains IMCC35004^T^ and IMCC35005^T^ from all the genera of the family *Desulfocapsaceae.*

Characteristics	1	2	3 (n = 2)	4 (n = 2)	5 (n = 2)	6 (n = 3)	7 (n = 1)
Cell shape	R	R	R	R	R	R	R
Motility by flagella	−	+	+	v	−	+	+
Oxidase	−	−	−	−	−^a^	ND	ND
Catalase	+	+	+	+	+^a^	ND	ND
Utilization of acetate	+	+	−	−	−	ND	−
Sulfite reduction	+	+	+	v	+	+	+
Elemental sulfur reduction	+	+	ND	−	−	+	+
Fe(III) citrate reduction	+	+	+	+	ND	+	ND
Quinone	MK-5	MK-5	MK-8(H_4_)	MK-6(H_2_)	MK-5(H_2_)	ND	MK-5
**Ranges for growth**							
Temperature (°C)	15–30	15–30	10–40	− 1.8–30	0–35	20–30	15–37
pH	6–9	6–8.5	7–9	6.5–9.5	5.7–8.2	6.8–8.0	6.7–8.3
NaCl (%)	1–6	2–5	1–9	1.5–5	0.5–5	1–1.5	ND
Major polar lipids	PE, PG, DPG	PE, PG	PE, PG	PE, PG, DPG	ND	ND	ND
Major fatty acids (> 10%)	C_17:1_ *ω*6*c*, C_16:0_, C_16:1_ *ω*5*c*	C_17:1_ *ω*6*c*, C_16:0_, SF3, C_16:1_ *ω*5*c*	C_16:0_, C_16:1_ *ω*5*c*, C_17:1_ *ω*6*c*	SF3, C_16:0_, C_14:0_	C_15:1_ *ω9c*, C_17:1_ *ω*8*c*,	ND	ND
Genome size (Mb)	5.7	6.8	6.1	3.5	5.0	4.0	5.0
Genomic G + C content (%)^a^	48.9	44.3	49.6, 50.3^b^	46.8	50.6	45.4	56.3

Strains: 1, IMCC35004^T^; 2, IMCC35005^T^; 3, *Desulfopila*^16,29^^; this study^; 4, *Desulfotalea*^15^^; this study^; 5, *Desulforhopalus*^14,30^; 6, *Desulfocapsa*^13,31^; 7, *Desulfofustis*^12^. n, number of species; + , positive; –, negative; v, variable; R, rod; ND, no data; PE, phosphatidylethanolamine; PG, phosphatidylglycerol; DPG, diphosphatidylglycerol; and SF3, summed feature 3 (C_16:1_ *ω*7*c* and/or C_16:1_ *ω*6*c*).

^a^Values calculated from the genomic data unless otherwise indicated.

^b^Value calculated using high-performance liquid chromatography.

**Figure 3:**
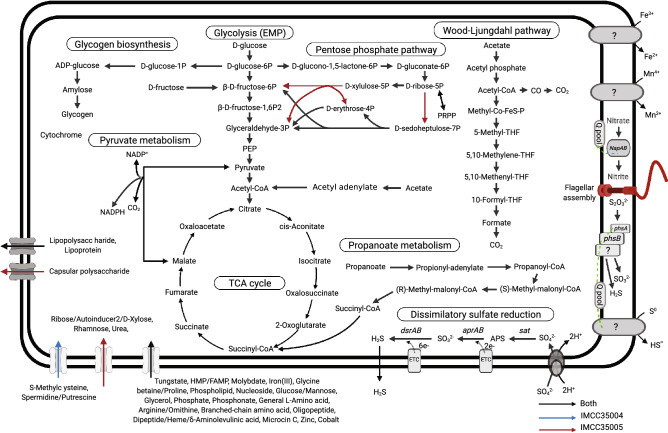
Overview of the metabolic pathways in strains IMCC35004^T^ and IMCC35005^T^. The presence and absence of genes were predicted based on the annotations from Kyoto Encyclopedia of Genes and Genomes. Genes predicted to be present in the phenotypic characterization but not found in the genome sequences are marked with question marks. Created with BioRender.com.

The genomes of strains IMCC35004^T^ and IMCC35005^T^ were found to contain genes for diverse metabolic pathways, as presented in Fig. 3 and Supplementary Table S2. In accordance with the experimental results, IMCC35004^T^ and IMCC35005^T^ genomes were identified to contain all the genes necessary for dissimilatory reduction of sulfate to hydrogen sulfide^32^. The two genomes contain multiple (4–5) copies of SulP family sulfate permease genes, transporting SO_4_^2−^ across the cell membrane. Additionally, the two genomes harbor the following genes for dissimilatory sulfate reduction: sulfate adenylyltransferase (*sat*), adenosine-5′-phosphosulfate reductase (*aprAB*), and dissimilatory sulfite reductase (*dsrAB*). Gene clusters of quinone-interacting membrane-bound oxidoreductase (*qmoABC*) and sulfite reduction-associated complex protein (*dsrMKJOP*) genes, which are involved in the electron transport from membrane-bound protein pools to cytoplasmic reductases (AprAB and DsrAB), were detected in each genome. The gene *dsrC*, which couples sulfate reduction to energy conservation^33^, and the gene *dsrD*, which is usually found in sulfite-reducing bacteria but not in sulfur-oxidizing bacteria^34^, were also predicted in both genomes. These genes related to dissimilatory sulfate reduction are also found in *Desulfopila* and *Desulfotalea* genomes.

The four SRB genomes employed in this study for the comparative genomic analyses were identified to contain the genes involved in the Embden-Meyerhof-Parnas pathway, tricarboxylic acid cycle, and dissimilatory sulfate and nitrate reduction, and also the genes encoding the ABC transporters for 4-amino-5-hydroxymethyl-2-methylpyrimidine (HMP)/*N*-formyl-4-amino-5-aminomethyl-2-methylpyrimidine (FAMP), tungstate, molybdate, iron(III), glycine betaine/proline, phospholipid, phosphate, general l-amino acids, branched-chain amino acids, lipoprotein, and lipopolysaccharide (Fig. 3 and Supplementary Table S2). However, IMCC35004^T^ and IMCC35005^T^ genomes differ from *Desulfopila* and *Desulfotalea* genomes in harboring genes involved in the Wood-Ljungdahl pathway or encoding acetaldehyde dehydrogenase (*adhE*), formate dehydrogenase (*fdhA*), lactoylglutathione lyase (*gloA*), sulfhydrogenase (*hydG*), sulfite dehydrogenase (*sorAB*), hydroxylamine dehydrogenase (*hao*), fumarylacetoacetase (*fahA*), and urocanate reductase (*urdA*). The gene clusters underlying flagellar assembly were detected in the genome of IMCC35005^T^ but not in IMCC35004^T^ genome, consistent with the transmission electron microscopy (TEM) results (Supplementary Fig. S3). The two genomes were predicted to harbor the genes related to the synthesis of phosphatidylglycerol (*pspA* and *cdsA*) and phosphatidylethanolamine (*psd* and *pspB*). In corroboration, two-dimensional thin-layer chromatography (TLC) of polar lipids yielded the corresponding spots (Supplementary Fig. S4).

Based on the COG functional profile, strains IMCC35004^T^ and IMCC35005^T^ were found to have high proportions of genes (> 5.0%) related to energy production and conversion (9.3–10.0% of all the COGs), signal transduction mechanisms (6.3–8.5%), amino acid transport and metabolism (6.5–6.9%), cell wall/membrane/envelope biogenesis (5.3–5.7%), general function prediction only (6.4–7.5%), and function unknown (5.1–5.2%), which are overall similar to those of the other two genomes from the family *Desulfocapsaceae* (Supplementary Table S3). Comparative genomic analyses using a Venn diagram (Supplementary Fig. S5) showed that the pan-genome of the four *Desulfocapsaceae* strains contains 1647 conserved core genes. A total of 664 and 708 genes from IMCC35004^T^ and IMCC35005^T^, respectively, were found to be unique.

### Phenotypic characteristics

The cells of strains IMCC35004^T^ and IMCC35005^T^ are Gram-stain-negative, chemoheterotrophic, strictly anaerobic, and rod-shaped. Both strains formed beige-colored, circular, convex, and smooth colonies with an entire margin and diameter of 0.4–0.7 mm after incubation on 3M-R2A at 25 °C for 3 weeks. The detailed phenotypic characteristics of strains IMCC35004^T^ and IMCC35005^T^ are provided in Tables 1, 2, 3, Supplementary Tables S4, and S5, and the protologues. Strains IMCC35004^T^ and IMCC35005^T^ differed in several phenotypic characteristics. The cells of strain IMCC35004^T^ were observed to be devoid of flagella, whereas those of strain IMCC35005^T^ harbored a polar flagellum (Supplementary Fig. S3), consistent with the results of the genomic analyses for the genes involved in flagella assembly. Both strains reduced sulfate, sulfite, elemental sulfur, thiosulfate, Fe(III) citrate, and Mn(IV) oxide but did not reduce nitrate or nitrite. Unlike strain IMCC35004^T^, strain IMCC35005^T^ reduced Fe(III) oxyhydroxide. Phenotypic characteristics of strains IMCC35004^T^ and IMCC35005^T^ were found to differ from those of other genera of the family *Desulfocapsaceae*, including the presence of flagella, growth range, and utilization of acetate as an electron donor, and sulfite as an electron acceptor (Table 1). Additionally, these two novel strains were found to differ from *Desulfopila aestuarii* DSM 18488^T^ and *Desulfotalea psychrophila* DSM 12343^T^ in hydrolysis of Tweens 20 and 80, enzyme activities, and carbon utilization patterns (Table 2, Supplementary Tables S4, and S5).

**Table 2:** Differential phenotypic characteristics of IMCC35004^T^, IMCC35005^T^, and the type strains of the related genera.

Characteristics	1	2	3	4
Cell size (width × length, µm)	0.7 − 0.9 × 2.2 − 2.6	0.9 − 1.1 × 1.5 − 2.2	0.7 − 1.2 × 1.9 − 3.8^a^	0.6 × 4.5 − 7.4^b^
Presence of flagella	−	+	+	+
**Growth range (optimum)**				
Temperature (°C)	15–30 (25)	15–30 (25)	10–40 (30)	4–18 (15) − 1.8–19 (10)^b^
NaCl (%)	1–6 (3)	2–5 (2)	1–5 (1) 0–5 (1)^a^	1–6 (1) 1.5–2.5 (1)^b^
pH	6.5–9 (7)	6–8.5 (7)	6.5–8.5 (7.5) 6.3–8.5 (7.5–7.6)^a^	6–8 (7) ND (7.3–7.6)^b^
**Electron acceptors**				
Nitrate	−	−	+	−
Fe(III) oxyhydroxide	−	+	+	−
**Electron donors**^**c**^				
Acetate	+	+	−	−
α-Ketoglutarate, alanine, betaine, ethanol, glycine, malate	+	−	−	−
Benzoate	−	−	+	−
Butanol, propanol, glycerol, succinate	−	+	−	−
Choline chloride, serine, mannitol	−	−	+	−
Glutamate, lactate	−	−	+	+
Propionate	−	−	−	+
**API ZYM**				
Esterase lipase (C8)	+	+	−	+
Leucine arylamidase	−	+	+	+
Valine arylamidase, β-galactosidase	−	−	−	+
*β*-Glucuronidase	−	+	−	+
α-Glucosidase	−	+	−	−
**Hydrolysis**				
Tween 20	+	−	+	−
Tween 80	+	−	−	−

Strains: 1, IMCC35004^T^; 2, IMCC35005^T^; 3, *Desulfopila aestuarii* DSM 18488^T^; 4, *Desulfotalea psychrophila* DSM 12343^T^. All the data are from this study unless otherwise indicated. All the strains are positive for reduction of sulfate, sulfite, thiosulfate, Fe(III) citrate and Mn(IV) oxide; production of H_2_S; catalase; utilization fumarate; alkaline phosphatase, esterase (C4), trypsin, acid phosphatase, and naphthol-AS-BI-phosphohydrolase. All the strains are negative for reduction of nitrite; utilization of butyrate or methanol; oxidase; lipase (C14); cystine arylamidase; *α*-chymotrypsin; *α*-galactosidase; *β*-glucosidase; *N*-acetyl-*β*-glucosaminidase; *α*-mannosidase; *α*-fucosidase; and hydrolysis of DNA, casein, CM-cellulose, and chitin. +, positive; –, negative, ND; no data.

^a^Data from Suzuki et al.^16^.

^b^Data from Knoblauch et al.^15^.

^c^Ethanol, butanol, and propanol were adjusted to 0.02%, benzoate was adjusted to 5 mM and other carbons to 20 mM.

**Table 3:** Cellular fatty acid compositions of IMCC35004^T^, IMCC35005^T^, and the type strains of the related genera.

Fatty acids (%)	1	2	3	4
**Saturated**				
C_14:0_	4.3	1.4	1.6	**14.3**
C_16:0_	**17.6**	**21.6**	**28.8**	**17.7**
C_17:0_	1.6	6.0	4.7	–
C_18:0_	6.0	Tr	Tr	Tr
**Unsaturated**				
C_14:1_ *ω*5*c*	–	–	Tr	2.7
C_15:1_ *ω*6*c*	–	4.0	4.0	2.9
C_16:1_ *ω*9*c*	1.1	Tr	–	–
C_16:1_ *ω*5*c*	**11.2**	**12.3**	**20.9**	9.2
C_17:1_ *ω*8*c*	3.0	1.7	–	–
C_17:1_ *ω*6*c*	**21.6**	**23.4**	**16.7**	Tr
C_18:1_ *ω*9*c*	3.6	Tr	–	3.2
C_18:1_ *ω*5*c*	2.4	Tr	Tr	–
Methyl C_18:1_ *ω*7*c*	1.2	Tr	Tr	–
**Branched**				
iso-C_14:0_	2.0	–	–	–
iso-C_15:0_	1.6	1.9	Tr	Tr
anteiso-C_15:0_	2.8	Tr	–	–
iso-C_16:0_ H	Tr	Tr	4.0	–
iso-C_16:0_	Tr	–	1.8	–
iso-C_17:0_	Tr	2.4	Tr	–
**Summed features**^**a**^				
3	6.1	**16.3**	**12.6**	**44.3**
4	–	2.6	1.1	–
5	–	Tr	–	1.2
8	9.8	Tr	Tr	Tr
9	–	1.8	Tr	–

Strains: 1, IMCC35004^T^; 2, IMCC35005^T^; 3, *Desulfopila aestuarii* DSM 18488^T^; 4, *Desulfotalea psychrophila* DSM 12343^T^. All the data are from this study. −, Not detected; Tr, traces (< 1.0%). Major fatty acids (> 10%) are shown in bold type.

^a^Summed features represent groups of two or three fatty acids which could not be separated using gas chromatography with the MIDI system. Summed feature 3 comprises C_16:1_ *ω*7*c* and/or C_16:1_ *ω*6*c*; Summed feature 4 comprises anteiso-C_17:1_ B and/or iso-C_17:1_ I; Summed feature 5 comprises C_18:2_ *ω*6,9*c* and/or anteiso-C_18:0_; Summed feature 8 comprises C_18:1_ *ω*7*c* and/or C_18:1_ *ω*6*c*; Summed feature 9 comprises 10-methyl C_16:0_ and/or iso-C_17:1_ *ω*9*c.*

The fatty-acid profiles of IMCC35004^T^, IMCC35005^T^, *Desulfopila aestuarii* DSM 18488^T^, and *Desulfotalea psychrophila* DSM 12343^T^ are shown in Table 3. The major cellular fatty acids (> 10%) of strain IMCC35004^T^ were C_17:1_ *ω*6*c* (21.6%), C_16:0_ (17.6%), and C_16:1_ *ω*5*c* (11.2%), and those of strain IMCC35005^T^ were C_17:1_ *ω*6*c* (23.4%), C_16:0_ (21.6%), summed feature 3 (C_16:1_ *ω*7*c* and/or C_16:1_ *ω*6*c*; 16.3%), and C_16:1_ *ω*5*c* (12.3%), showing different proportions from those of the related type species. For example, although C_17:1_ *ω*6*c* is highly found in strains IMCC35004, IMCC35005, and DSM 18488^T^, constituting 16.7–23.4% of the total cellular fatty acids, it was not detected in the genus *Desulfotalea.*

Strain IMCC35004^T^ contains phosphatidylethanolamine (PE), phosphatidylglycerol (PG), and diphosphatidylglycerol (DPG) as the major polar lipids, whereas IMCC35005^T^ contains only PE and PG. These two polar lipids are consistently found in the genera *Desulfopila* and *Desulfotalea,* but the presence of DPG among the genera varies (Table 1). The predominant isoprenoid quinone detected in strains IMCC35004^T^ and IMCC35005^T^ is menaquinone-5 (MK-5), whereas those of the genera *Desulfopila* and *Desulfotalea* are MK-8 and MK-6, respectively (Table 1), suggesting that the two strains may belong to a novel genus from the family *Desulfobulbaceae*.

### Bacterial community structure of the tidal-flat sediment

**Figure 4:**
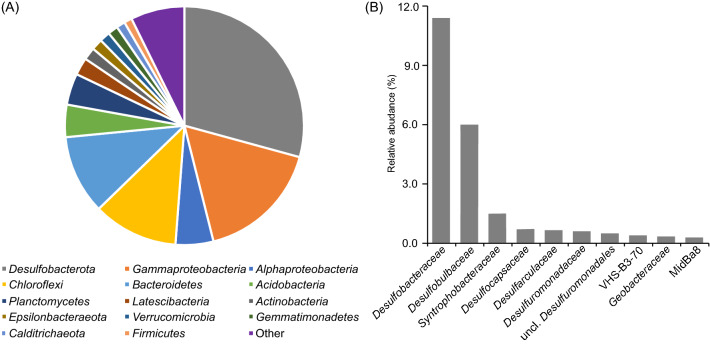
Relative abundance of bacterial taxa at the phylum/class level (**a**) and the 10 most abundant families from the phylum *Desulfobacterota* (**b**).

The bacterial community of the tidal-flat sediment sample from which strains IMCC35004^T^ and IMCC35005^T^ were isolated was analyzed using 16S rRNA gene amplicon sequencing. Classification of the resulting total of 113,202 reads showed that the tidal-flat sediment sample harbored diverse bacterial taxa (Fig. 4). At the phylum/class level, the phylum *Desulfobacterota* (28.4%; 16S rRNA gene sequence abundance) and the class *Gammaproteobacteria* (16.8%) were identified to be the predominant bacteria, followed by the phyla *Chloroflexi* (11.5%) and *Bacteroidetes* (10.7%). This bacterial community structure is similar to those previously reported for tidal-flat sediments^20,35^. The most abundant family detected from the phylum *Desulfobacterota* was *Desulfobacteraceae* (11.4%), followed by the families *Desulfobulbaceae* (6.0%), *Syntrophobacteraceae* (1.5%), and *Desulfocapsaceae* (0.7%), indicating that SRB were enriched in the sampling area. To determine the relative abundance of the bacterial phylotypes related to strains IMCC35004^T^ and IMCC35005^T^, the number of 16S rRNA gene amplicon sequences showing ≥ 98.7% sequence similarity to those of the isolates were determined using local BLAST search. The numbers of the amplicon sequences corresponding to the phylotypes related to IMCC35004^T^ and IMCC35005^T^ were 519 (0.46% of total) and 538 (0.48%), respectively, indicating that the isolate-related phylotypes in the tidal flat constitute a minor portion of the bacterial community.

### Taxonomic conclusion

Low 16S rRNA gene sequence similarity, low values of ANI and dDDH, and distinct phenotypic differences between strains IMCC35004^T^ and IMCC35005^T^ indicated that the two strains represent two independent species. However, inconsistencies in the phylogenetic positions of the two strains between the phylogenetic tree based on the 16S rRNA genes and that based on the whole genomes makes it difficult to assign the two strains into specific genera. Based on the AAI and POCP values between the two strains and the genera *Desulfopila* and *Desulfotalea*, the two strains might marginally belong to the genus *Desulfopila* or a novel genus, but formation of a robust phylogenetic clade and the difference in respiratory quinone content justify the classification of the two strains into a novel genus. Since physiological, genomic, and chemotaxonomic analyses showed that the two strains are novel sulfate-reducing bacteria distinct from the other type species in the family *Desulfocapsaceae,* the names *Desulfosediminicola ganghwensis* gen. nov., sp. nov. and *Desulfosediminicola flagellatus* sp. nov. are proposed, with IMCC35004^T^ and IMCC35005^T^ as the type strains, respectively.

### Description of *Desulfosediminicola* gen. nov.

*Desulfosediminicola* (De.sul.fo.se.di.mi.ni′co.la. L. pref. *de* from; L. neut. n. *sulfur* sulfur; L. neut. n. *sedimen* sediment; L. masc. suff. -*cola* (from L. masc. n. *incola*) an inhabitant; N.L. masc. n. *Desulfosediminicola* sulfate reducing sediment dweller).

Gram-stain-negative, sulfate-reducing, strictly anaerobic, and chemoheterotrophic. Rod-shaped. Oxidase-negative and catalase-positive. Sulfate, sulfite, elemental sulfur, thiosulfate, Fe(III) citrate, and Mn(IV) oxide are reduced as electron acceptors. Requires NaCl for growth. The major fatty acids are C_17:1_ *ω*6*c*, C_16:0_, and C_16:1_ *ω*5*c*. The major quinone is MK-5. The major polar lipids identified are PE and PG. Belongs to the family *Desulfocapsaceae*. The type species is *Desulfosediminicola ganghwensis*.

### Description of *Desulfosediminicola ganghwensis* sp. nov.

*Desulfosediminicola ganghwensis* (gang.hwen′sis. N.L. masc. adj. *ganghwensis* pertaining to Ganghwa Island, Republic of Korea, the geographical origin of the type strain of the species).

Gram-stain-negative, strictly anaerobic, and rod-shaped with a width of 0.7–0.9 μm and length of 2.2–2.6 μm. The colonies are 0.4–0.7 mm in diameter, beige-colored, smooth, convex, and circular with an entire margin after 3 weeks of incubation on 3M-R2A at 25 °C. Black colonies emerge on MA. Requires vitamins for growth. Growth occurs at 15–30 °C (optimum, 25 °C) and pH 6.5–9.0 (optimum, pH 7.0) with 1.0–6.0% NaCl (optimum, 3.0%). Tween 20 and Tween 80 are hydrolyzed, but starch, CM-cellulose, chitin, casein, or DNA is not hydrolyzed. Sulfate, sulfite, elemental sulfur, thiosulfate, Fe(III) citrate, and Mn(IV) oxide are used as electron acceptors, but Fe(III) oxyhydroxide, nitrate, or nitrite is not reduced. No autotrophic growth based on thiosulfate disproportionation or hydrogen oxidation is observed. H_2_S is produced. *α*-Ketoglutarate, acetate, alanine, betaine, ethanol, fumarate, glycine, and malate are used as electron donors, but benzoate, butanol, butyrate, choline chloride, glutamate, glycerol, lactate, mannitol, methanol, propanol, propionate, serine, or succinate is not utilized. In API 20A, acid is produced from salicin, d-xylose, glycerol, d-mannose, and d-melezitose but not from d-glucose, d-mannitol, d-lactose, d-saccharose, d-maltose, l-arabinose, d-cellobiose, d-raffinose, d-sorbitol, l-rhamnose, or d-trehalose. Hydrolysis of esculin is positive, but gelatin liquefaction is negative. Indole formation and urease are undetected. In API ZYM, alkaline phosphatase, esterase (C4), esterase lipase (C8), acid phosphatase, and naphthol-AS-BI-phosphohydrolase were detected, but lipase (C14), leucine arylamidase, valine arylamidase, cystine arylamidase, trypsin, α-chymotrypsin, *α*-galactosidase, *β*-galactosidase,* β*-glucuronidase, *α*-glucosidase, *β*-glucosidase, *N*-acetyl-*β*-glucosaminidase, *α*-mannosidase, or *α*-fucosidase was undetected. The respiratory quinone is MK-5. The major fatty acids are C_17:1_ *ω*6*c*, C_16:0_ and C_16:1_ *ω*5*c*. The major polar lipids are PE, PG, and DPG, an unidentified aminophospholipid, two unidentified aminolipids, four unidentified phospholipids, and four unidentified lipids. The type strain IMCC35004^T^ (= KCTC 15826^T^ = NBRC 114003^T^) was isolated from the tidal-flat sediment of Ganghwa Island, Republic of Korea. The whole-genome of the type strain is 5.7 Mb. The genomic G + C content of the type strain is 48.9 mol%. The GenBank accession numbers of the 16S rRNA gene sequences and the complete genome sequence of the type strain are MK660563 and CP05699, respectively.

### Description of *Desulfosediminicola flagellatus* sp. nov.

*Desulfosediminicola flagellatus* (fla.gel.la′tus. L. part. adj. *flagellatus* flagellated).

The cells are Gram-stain-negative, strictly anaerobic, motile by a single polar flagellum, and rod-shaped with a width of 0.9–1.1 μm and length of 1.5–2.2 μm. The colonies are 0.4–0.7 mm in diameter, beige-colored, smooth, convex, and circular with an entire margin after 3 weeks of incubation on 3M-R2A at 25 °C. Black colonies emerge on MA. Requires vitamins for growth. Growth occurs at 15–30 °C (optimum, 25 °C) and pH 6.0–8.5 (optimum, pH 7.0) with 2.0–5.0% NaCl (optimum, 2.0%). Starch, CM-cellulose, chitin, casein, Tween 20, Tween 80, or DNA is not hydrolyzed. Sulfate, sulfite, elemental sulfur, thiosulfate, Fe(III) citrate, Fe(III) oxyhydroxide, and Mn(IV) oxide are used as electron acceptors, but nitrate or nitrite is not reduced. No autotrophic growth based on thiosulfate disproportionation or hydrogen oxidation is observed. H_2_S is produced. Acetate, butanol, fumarate, glycerol, propanol, and succinate are used as electron donors, but α-ketoglutarate, alanine, benzoate, betaine, butyrate, choline chloride, ethanol, glutamate, glycine, lactate, malate, mannitol, methanol, propionate, or serine is not utilized. In API 20A, acid is produced from d-glucose, d-mannitol, d-saccharose, d-maltose, salicin, l-arabinose, esculin, glycerol, d-cellobiose, d-sorbitol, or d-trehalose but not from d-lactose, d-xylose, d-mannose, d-melezitose, d-raffinose, or l-rhamnose. Hydrolysis of esculin is positive, but gelatin liquefaction is negative. Indole formation and urease are undetected. In API ZYM, alkaline phosphatase, esterase (C4), esterase lipase (C8), leucine arylamidase, acid phosphatase, naphthol-AS-BI-phosphohydrolase, *β*-glucuronidase, and α-glucosidase are detected, but lipase (C14), valine arylamidase, cystine arylamidase, trypsin, *α*-chymotrypsin, *α*-galactosidase, *β*-galactosidase, *β*-glucosidase, *N*-acetyl-*β*-glucosaminidase, α-mannosidase, or α-fucosidase is not. The predominant respiratory quinone is MK-5. The major fatty acids are C_17:1_ *ω*6*c*, C_16:0_, summed feature 3 (C_16:1_ *ω*7*c* and/or C_16:1_ *ω*6*c*), and C_16:1_ *ω*5*c*. The major polar lipids are phosphatidylethanolamine, phosphatidylglycerol, an unidentified aminolipid, and five unidentified lipids. The type strain IMCC35005^T^ (= KCTC 15827^T^ = NBRC 114004^T^) was isolated from the tidal-flat sediment of Ganghwa Island, Republic of Korea. The whole-genome of the type strain is 6.8 Mb. The genomic G + C content of the type strain is 44.3 mol%. The GenBank accession numbers of the 16S rRNA gene sequence and the complete genome sequence of the type strain are MK660564 and CP05698, respectively.

## Materials and methods

### Isolation and culture conditions

A sediment sample was collected off the coast of Ganghwa Island (37° 35′ 26″ N, 126° 27′ 17″ E), South Korea, in July 2018 using a 30 cm long acrylic core sampler with a 10 cm diameter. The sediment core was sealed in an anaerobic bag, transported to the laboratory in an ice cooler, and then transferred into a vinyl anaerobic chamber (Coy Laboratory Products) filled with N_2_:H_2_:CO_2_ (90:5:5). A sub-sample (1 g) was collected from the middle of the sediment core and homogenized with 10 ml of sterile aged seawater. For long-term storage, 500 μl of the diluted homogenized sample in 10% (v/v) glycerol solution was stored at − 80 °C. An aliquot (100 μl) of the diluted homogenized sediment sample was spread onto marine agar 2216 (MA; BD Diagnostics) and anaerobically incubated in an anaerobic jar (Mitsubishi Gas Chemical) at 17 °C. After incubation for 3 weeks, two black-colored colonies that appeared on the MA plates were picked and sub-cultured three times. After 16S RNA gene sequencing, the two strains were designated as IMCC35004^T^ and IMCC35005^T^. After the optimum culture conditions had been determined, the two strains were routinely maintained on MA or 3M-R2A (0.5 g yeast extract, 0.5 g casamino acid, 0.5 g proteose peptone no.3, 0.5 g glucose, 0.5 g soluble starch, 0.3 g sodium pyruvate, and 15 g agar in 1 L of 80% aged seawater) at 25 °C under anaerobic conditions. The cultures were stored at − 80 °C as 10% (v/v) glycerol suspensions. For physiological and chemotaxonomic comparisons, the type species *Desulfopila aestuarii* DSM 18488^T^ and *Desulfotalea psychrophila* DSM 12343^T^ were obtained from the Deutsche Sammlung von Mikroorganismen und Zellkulturen GmbH (DSMZ). *Desulfopila aestuarii* DSM 18488^T^ and *Desulfotalea psychrophila* DSM 12343^T^ were routinely cultivated at 25 °C and 15 °C, respectively, on MA or 3M-R2A under anaerobic conditions. Unless otherwise indicated, all the media used in this study were autoclaved in a Duran bottle equipped with a rubber stopper, after sparging with N_2_ gas for 20 min per 1 L medium.

### Phylogenetic analyses based on 16S rRNA gene sequences

The genomic DNAs of strains IMCC35004^T^ and IMCC35005^T^ were extracted using DNeasy Blood and Tissue kit (Qiagen) according to the manufacturer’s instructions. The 16S rRNA genes of the strains were amplified using PCR with the bacterial universal primers 27F and 1492R^36^ and sequenced using a Sanger sequencer. The resultant almost complete 16S rRNA gene sequences of strains IMCC35004^T^ (1469 bp) and IMCC35005^T^ (1470 bp) were identified using BLASTn at GenBank and the “16S-based ID service” in the EzBioCloud database^17^. For phylogenetic analyses, the 16S rRNA gene sequences of the two strains were aligned using the SILVA Incremental Aligner^37^ and imported into the ARB database^38^. The aligned and manually curated 16S rRNA gene sequences in the ARB database were exported to the MEGA X program^39^, which was then used for constructing phylogenetic trees based on the neighbor-joining^40^, maximum-likelihood^41^, and minimum-evolution^42^ methods with Jukes-Cantor parameter, the Tamura-Nei model, and Jukes-Cantor correction, respectively. The robustness of the phylogenetic trees was evaluated using bootstrap analyses based on 1000 random re-samplings^43^.

### Sequencing of the 16S rRNA gene amplicons derived from the tidal-flat sediment sample

For 16S rRNA gene amplicon sequencing of the sediment sample, the diluted homogenized sample stored at − 80 °C was thawed and subsequently centrifuged at 10,000*g* for 1 h. After the supernatant was removed, DNA was extracted from the sediment pellet by using the DNeasy PowerSoil Kit (Qiagen) following the manufacturer’s instructions. The quantity and quality of the DNA were measured using Qubit 3.0 (ThermoFisher Scientific) and Nanodrop (ThermoFisher Scientific). The V4 region of the 16S rRNA gene was amplified using fusion primers that were designed based on the universal primers 515F-Y and 806R-B^44^. Amplicon sequencing of pooled PCR products was performed using an Illumina MiSeq System (2 × 250 bp) at ChunLab, Inc. (Korea). The raw amplicon sequencing data were processed using the Quantitative Insights into Microbial Ecology Version 2 (QIIME2, version 2019.10.)^45^. Primer sequences were removed using Cutadapt^46^, and DADA2 plugin was used for quality-filtering, read-joining, chimera removal, and denoising^47^. The amplicon sequence variants (ASVs) obtained using DADA2 were classified using the “feature-classifier” command (with the “classify-sklearn” option) and a classifier pre-trained based on Silva 138 release (“silva-138-99-515-806-nb-classifier.qza”).

### Whole-genome sequencing and genomic analyses

Whole-genome sequencing of strains IMCC35004^T^ and IMCC35005^T^ was performed by both Illumina and Nanopore platforms. For Illumina sequencing, the genomic DNAs of strains IMCC35004^T^ and IMCC35005^T^ were extracted using DNeasy Blood and Tissue kit (Qiagen) according to the manufacturer’s instructions. DNA library was constructed using the TruSeq Nano DNA Library Prep Kit (Illumina). Illumina sequencing was performed at ChunLab, Inc. (Korea) by using the MiSeq system with 2 × 300 bp paired-end run. For Nanopore genome sequencing, the genomic DNAs were extracted using the MagAttract HMW DNA Kit (Qiagen). Sequencing libraries were prepared using the Ligation Sequencing Kit (SQK-LSK109, Oxford Nanopore Technologies) and Native Barcoding Expansion pack (EXP-NBD104, Oxford Nanopore Technologies) and then loaded and sequenced using a flow cell (R9.4.1) on a MinION device (Oxford Nanopore Technologies). Guppy (v3.4.5) was used at the high-accuracy mode for the base-calling of the nanopore data. The hybrid assemblies of the both Illumina and Nanopore reads were performed using Unicycler version 0.4.8^48^. The following *Desulfocapsaceae* genomes were downloaded from the GenBank for the genomic comparison: *Desulfopila aestuarii* DSM 18488^T^ (FRFE00000000) and *Desulfotalea psychrophila* LSv54^T^ (CR522870). For calculating the genome relatedness among the *Desulfocapsaceae* genomes, the corresponding ANI and dDDH values were calculated using the JSpecies web server^49^ and genome-to-genome distance calculator (GGDC 2.1)^50^. The AAI^51^ and POCP^27^ were computed to measure the genomic similarity based on the amino-acid sequences. Phylogenomic tree was inferred using the up-to-date bacterial core gene (UBCG) pipeline, consisting of 92 genes, with the default parameters^52^.

For the reconstruction of metabolic pathways, each genome was annotated using Prokka^53^, and the resultant open reading frames were queried into BlastKOALA^54^ and KofamKOALA^55^ according to the Kyoto Encyclopedia of Genes and Genomes database. For the analysis of distribution of Clusters of Orthologous Groups (COG) categories, the protein sequences obtained using Prokka were queried against the COG database by using RPS-BLAST (e-value cutoff; 0.01)^56^. The orthogroups among the genome-encoded proteins were determined using OrthoFinder (version 2.3.11) with BLAST + as a sequence search program^57^, and the number of shared or unique orthologous protein clusters was depicted on a Venn diagram by using the package “venn” (version 1.9) in R environment.

### Morphological, physiological, and biochemical characterization

As for phenotypic characterization, unless otherwise noted, cells grown on MA or in MB (marine broth 2216) at 25 °C for 3 weeks under anaerobic conditions were used. Gram-staining was performed using a Gram-staining kit (bioMérieux). Cell morphology was examined using a transmission electron microscope (TEM; CM200, Philips). A TEM specimen was prepared using a carbon-coated copper grid (Electron Microscopy Sciences) holding the cells stained with 2% (w/v) uranyl acetate. Motility by flagella was confirmed using the semi-solid R2A agar medium (0.5%). Growth was measured at 4, 10, 15, 20, 25, 30, 37, 42, and 45 °C in MB. The pH range and optimal value for growth were monitored in MB adjusted to pH 5.0–10.0 (at 1.0 pH unit intervals) by using the following buffers: 1 M MES, 1 M MOPS, 1 M HEPES, 1 M Tris, and 0.5 M CHES buffers for pH 5.0–6.0, 7.0, 8.0, 9.0, and 10.0, respectively. The growth at various concentrations of NaCl (0–8.0% at increments of 1.0%) were determined by supplementing NaCl into NaCl-free MB. Catalase and oxidase activities were assessed using 3.0% (v/v) hydrogen peroxide and the Kovac’s solution (bioMérieux), respectively. The bacterial strains were tested for their abilities to hydrolyze the following macromolecules, which were added into 3M-R2A: casein (3.0% skimmed milk, w/v), chitin (1.0%, w/v), starch (1.0%, w/v), CM-cellulose (1.0% CM-cellulose, w/v), Tween 20 (1.0%, w/v), or Tween 80 (1.0%, w/v). DNase test agar (BD Diagnostics) supplemented with 2% NaCl was used to assess for DNA-degradation activity. Production of H_2_S was investigated using triple sugar iron agar (TSI; BD Diagnostics) with 2% NaCl.

The electron acceptors for the anaerobic respiration were evaluated in a modified artificial seawater (ASW) medium (19.45 g NaCl, 8.8 g MgCl_2_, 1.8 g CaCl_2_, 0.55 g KCl, 0.16 g NaHCO_3_, 0.08 g KBr, 0.034 g SrCl_2_, 0.022 g H_3_BO_3_, 0.0024 g NaF, 0.004 g Na_2_SiO_3_, 0.0016 g NH_4_NO_3_, and 0.008 g Na_2_HPO_4_ in 1 L deionized water) containing trace metals^58^ and a vitamin mixture^59^. The following electron acceptors were tested in the ASW medium supplemented with 20 mM acetate as the electron donor: sulfate (20 mM Na_2_SO_4_), sulfite (10 mM Na_2_SO_3_), elemental sulfur (powder; 1 mM), thiosulfate (10 mM Na_2_S_2_O_3_), Fe(III) citrate (5 mM FeC_6_H_5_O_7_), Fe(III) oxyhydroxide [5 mM Fe(OH)_3_], Mn(IV) (345 µM MnO_2_), nitrate (3 mM NaNO_3_), and nitrite (484 µM NaNO_2_). Cells (10^4^ cells/ml) were inoculated into 10 ml ASW medium in a serum bottle and cultivated at 25 °C for 3 weeks. The concentrations of sulfate, nitrate, and nitrite were determined using the SulfaVer 4 (Hach), cadmium-reduction (Hach), and ferrous-sulfate (Hach) methods, respectively, according to the manufacturer’s instructions. Reduction of sulfite and elemental sulfur, Fe(III), and Mn(IV) was determined by measuring the concentrations of sulfide, Fe(II), and Mn(II) by using the methylene-blue method (Hach), 1,10-Phenanthroline Kit (Hach), and WAK-Mn test (Pack Test kits; Kyoritsu Chemical-Check Lab), respectively. Reduction of thiosulfate was determined based on the colorimetric method described previously^60^. To evaluate the electron donors, 19 sole-carbon sources at a final concentration of 20 mM or 0.02% (v/v), except for benzoate (5 mM), were added into the ASW medium with 20 mM of Na_2_SO_4_ as the electron acceptor. After incubation at 25 °C for 3 weeks, the utilization of the electron donors was determined by the presence of sulfide^15^ and the cellular growth measured using an Easy-Cyte flow cytometer (Guava Technologies) after staining with 1: 2000 (v/v)-diluted SYBR-Green I (Invitrogen). Thiosulfate disproportionation was tested in the ASW medium supplemented with 10 mM of thiosulfate and the growth was monitored after 1, 2, and 3 weeks of incubation using the flow cytometer. Hydrogen-dependent autotrophic growth was tested using the ASW medium under the ambient hydrogen concentration (N_2_:H_2_:CO_2_ = 90:5:5) in the anaerobic chamber, and the growth was measured using the flow cytometer after incubation at 25 °C for 3 weeks. Additional biochemical tests were performed using API 20A, API ZYM, and API 50CH test strips (bioMérieux) according to the instructions of the manufacturers except that ASW was used as the suspension medium for the API ZYM test and cysteine as the reducing agent for the API 50CH medium. The results of the tests were recorded after 2 weeks of incubation at 25 °C under anaerobic conditions.

### Chemotaxonomic characterization

The fatty-acid methyl ester (FAME) profiles of the bacteria were determined via gas chromatography (Agilent 7890A) by using Sherlock Microbial Identification System version 6.1 (MIDI) with the TSBA6 database according to the manufacturer’s protocol^61^. For FAME analysis, the cells of IMCC35004^T^, IMCC35005^T^, *Desulfopila aestuarii* DSM 18488^T^, and *Desulfotalea psychrophila* DSM 12343^T^ were harvested from colonies grown on the same sectors of the plates after anaerobic incubation on MA at 25 °C (15 °C for *Desulfotalea psychrophila* DSM 12343^T^) for 3 weeks. Polar lipids were extracted according to the protocol described by Minnikin et al.^62^ and separated using two-dimensional TLC on silica-gel 60 F_254_ plates (Merck). All the polar lipids on the TLC plates were visualized by spraying the plates with molybdatophosphoric acid. The aminolipids, phospholipids, and glycolipids on replicate TLC plates were identified by spraying the plates with ninhydrin, molybdenum blue, and alpha-naphthol solution, respectively. Respiratory isoprenoid quinones were extracted^62^ and examined via reverse-phase partition chromatography by using HPTLC RP-18F_254_ (Merck), according to the method described by Collins et al.^63^.

### Nucleotide sequence accession numbers

The 16S rRNA gene sequences and the whole-genome sequences of strains IMCC35004^T^ and IMCC35005^T^ were deposited in GenBank/EMBL/DDBJ under the accession numbers MK660563 and MK660564, and CP050699 and CP050698, respectively.

## Supplementary Information

Supplementary Information.
