# Supplementary material for: A sulfate-reducing bacterial genus, Desulfosediminicola gen. nov., comprising two novel species cultivated from tidal-flat sediments
Source: Sci Rep. 2021 Oct 7;11:19978. doi: 10.1038/s41598-021-99469-5 (PMC8497536; doi:10.1038/s41598-021-99469-5)

Supplementary Materials

Table S1. Values of AAI, POCP, ANI, and dDDH between pairs of genomes of IMCC35004^T, IMCC35005^T, and closely related genera of the family *Desulfocapsaceae*.

Strains: 1, IMCC35004^T; 2, IMCC35005^T; 3, *Desulfopila aestuarii* DSM 18488^T; 4, *Desulfotalea psychrophila* LSv54^T.

	AAI (%)				POCP (%)				ANI (%)				dDDH (%)			
	1	2	3	4	1	2	3	4	1	2	3	4	1	2	3	4
1		68.6	67.3	57.0		64.9	47.9	43.8		70.2	70.1	67.4		20.5	20.7	24.4
2			69.0	56.8			49.6	42.9			70.0	67.5			19	23.9
3				56.4				37.5				67.5				20.3

Table S2. Genome features of IMCC35004^T, IMCC35005^T, and two related type strains in the family *Desulfocapsaceae*.

Strains: 1, IMCC35004^T; 2, IMCC35005^T; 3, *Desulfopila aestuarii* DSM 18488^T; 4, *Desulfotalea psychrophila* LSv54^T.

**Characteristics**	**1**	**2**	**3**	**4**
Accession number	CP050699	CP050698	FRFE00000000	CR522870
Genome size (bp)	5,653,142	6,751,878	6,065,581	3.523,383
DNA G+C content (mol%)	48.4	44.3	49.6	46.8
Contig(s)	1	1	103	1
Protein-coding genes	4,715	5,479	5,044	3,051
tRNA	73	76	49	65
rRNA	18	21	9	21
5S rRNA	6	7	3	7
16S rRNA	6	7	3	7
23S rRNA	6	7	3	7
Presence/absence of genes				
Acetaldehyde dehydrogenase, formate dehydrogenase, lactoylglutathione lyase, lactate racemase, sulfhydrogenase, sulfite dehydrogenase, hydroxylamine dehydrogenase, fumarylacetoacetase, urocanate reductase	+	+	−	−
Wood-Ljungdahl pathway, acetoacetyl-CoA synthetase, sulfide:quinone oxidoreductase, anaerobic carbon-monoxide dehydrogenase, nicotinamidase, lactate racemase, sucrose synthase	+	+	+	−
α-Galactosidase	+	−	+	−
Xylose isomerase	−	+	+	−
Flagellar assembly	−	+	+	+
Butyrate kinase, nitrite reductase, altronate hydrolase	−	−	+	−
Galactonate dehydratase	−	+	−	−
ABC transporters for				
Spermidine/putrescine	+	−	+	−
Arginine/ornithine	+	+	−	−
D-Xylose, erythritol	−	−	+	−
Rhamnose, urea, capsular polysaccharide	−	+	−	−
Molybdate/tungstate, glutamine	−	−	−	+
Glycerol, phosphonate, microcin C, lipopolysaccharide	+	+	+	−
S-methylcysteine	+	−	−	−

Table S3. Distribution of genes based on COG functional categories in the genomes of strains IMCC35004^{T}, IMCC35005^{T}, and the most closely related species of the family *Desulfocapsaceae*.

Strains: 1, IMCC35004^{T}; 2, IMCC35005^{T}; 3, *Desulfopila aestuarii* DSM 18488^{T}; 4, *Desulfotalea psychrophila* LSv54^{T}. The number exhibited percentages of genes in each COG group. -, not detected.

Characteristics		1	2	3	4
J	Translation	4.9	4.6	4.7	7.3
A	RNA processing and modification	0.1	–	–	–
K	Transcription	3.9	3.9	4.1	3.2
L	Replication, recombination and repair	3.2	2.5	2.6	3.1
B	Chromatin structure and dynamics	0.1	0.1	0.1	0.1
D	Cell cycle control, cell division, chromosome partitioning	1.0	1.1	1.5	1.9
V	Defense mechanisms	1.5	1.6	1.5	1.6
T	Signal transduction mechanisms	6.3	8.5	9.8	5.5
M	Cell wall/membrane/envelope biogenesis	5.3	5.7	5.0	5.5
N	Cell motility	1.3	2.8	2.8	3.5
U	Intracellular trafficking, secretion, and vesicular transport	1.1	1.0	1.0	1.4
O	Posttranslational modification, protein turnover, chaperones	3.7	3.7	3.2	3.5
X	Mobilome: prophages, transposons	2.0	1.0	1.2	0.6
C	Energy production and conversion	10.0	9.3	9.5	8.0
G	Carbohydrate transport and metabolism	5.3	4.7	5.1	3.6
E	Amino acid transport and metabolism	6.9	6.5	6.7	7.3
F	Nucleotide transport and metabolism	1.7	1.6	1.7	2.2
H	Coenzyme transport and metabolism	3.8	3.5	3.4	4.9
I	Lipid transport and metabolism	3.5	3.2	3.5	2.7
P	Inorganic ion transport and metabolism	3.9	3.6	4.0	4.4
Q	Secondary metabolites biosynthesis, transport and catabolism	1.4	1.9	1.3	1.1
R	General function prediction only	6.4	7.5	6.0	6.3
S	Function unknown	5.1	5.2	5.3	6.0

Table S4. Physiological characteristics (API 20A) of strains IMCC35004^T, IMCC35005^T, and closely related type strains of the family *Desulfocapsaceae*.

Strains: 1, IMCC35004^T; 2, IMCC35005^T; 3, *Desulfopila aestuarii* DSM 18488^T; 4, *Desulfotalea psychrophila* DSM 12343^T. All data were obtained in this study. +, positive; -, negative.

Characteristics	1	2	3	4
Indole formation	−	−	−	−
Urease	−	−	+	−
Acid production from				
D-Glucose	+	+	−	+
D-Mannitol	−	+	−	−
D-Lactose	−	−	−	−
D-Saccharose	−	+	+	−
D-Maltose	+	+	−	−
Salicin	+	+	−	+
D-Xylose	+	−	+	−
L-Arabinose	−	+	−	−
Glycerol	+	+	+	−
D-Cellobiose	−	+	+	+
D-Mannose	+	−	−	+
D-Melezitose	+	−	+	−
D-Raffinose	−	−	+	−
D-Sorbitol	−	+	−	−
L-Rhamnose	−	−	+	−
D-Trehalose	+	+	+	+
Hydrolysis of				
Gelatin	+	−	−	−
Esculin	+	+	+	−

Table S5. Acid production pattern (API 50CH) of strains IMCC35004^T, IMCC35005^T, and closely related type strains of the family *Desulfocapsaceae*.

Strains: 1, IMCC35004^T; 2, IMCC35005^T; 3, *Desulfopila aestuarii* DSM 18488^T; 4, *Desulfotalea psychrophila* DSM 12343^T. All data were obtained in this study. +, positive; -, negative.

**Acid production from**	**1**	**2**	**3**	**4**
Glycerol	−	−	−	−
Erythritol	−	−	−	−
D-Arabinose	−	−	−	−
L-Arabinose	−	−	−	−
D-Ribose	−	−	−	−
D-Xylose	−	−	−	−
L-Xylose	−	−	−	−
D-Adonitol	−	−	−	−
Methyl-*β*-D-xyloside	−	−	−	−
D-Galactose	−	−	−	−
D-Glucose	−	+	+	−
D-Fructose	−	+	+	+
D-Mannose	−	−	−	−
L-Sorbose	−	−	−	−
L-Rhamnose	−	−	−	−
Dulcitol	−	−	−	−
Inositol	−	−	−	−
D-Mannitol	−	−	−	−
D-Sorbitol	−	−	−	−
Methyl-*α*-D-mannopyranoside	−	−	−	−
Methyl-*α*-D-glucopyranoside	−	−	−	−
N-acetyl-glucosamine	−	−	−	−
Amygdalin	−	−	−	−
Arbutin	−	−	−	−
Esculin	+	+	+	+
Salicin	−	−	−	−
D-Cellobiose	−	−	−	−
D-Maltose	−	+	−	−
D-Lactose	−	+	+	−
D-Melibiose	−	+	+	+
D-Saccharose	−	−	−	−

D-Trehalose	−	−	−	−
Inulin	−	−	−	−
D-Melezitose	−	−	−	−
D-Raffinose	−	−	−	−
Starch	−	−	−	−
Glycogen	−	−	−	−
Xylitol	−	−	−	−
Gentiobiose	−	−	−	−
D-Turanose	−	−	−	−
D-Lyxose	−	−	−	−
D-Tagatose	−	−	−	−
L-Fucose	−	−	−	−
D-Arabitol	−	−	−	−
L-Arabitol	−	−	−	−
Gluconate	−	−	−	−
2-Keto-gluconate	−	−	−	−
5-Keto-gluconate	−	−	−	−

Fig. S1. Circular map of the IMCC35004^{T} chromosome. From outside to the center: RNA genes (tRNAs brown, rRNAs purple; other RNAs grey), GC content, GC skew.

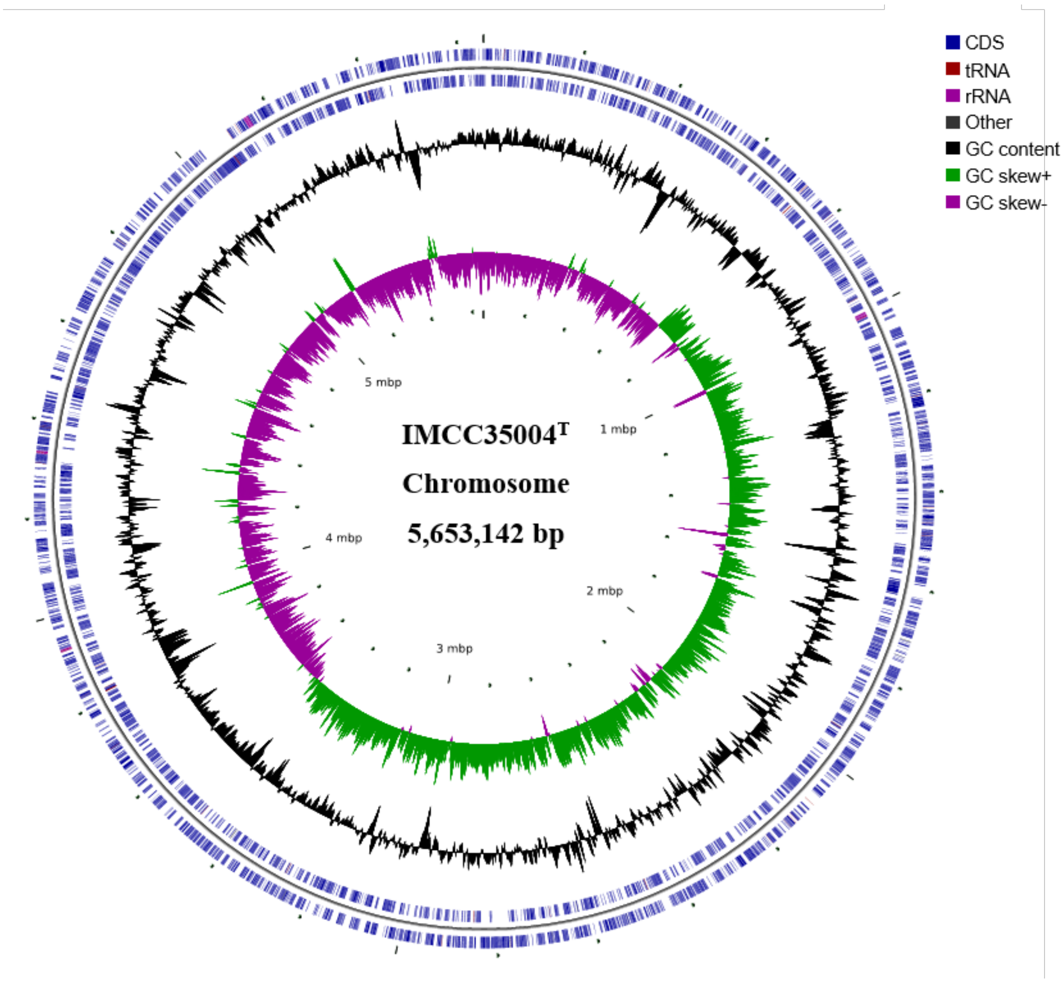

Fig. S2. Circular map of the IMCC35005^{T} chromosome. From outside to the center: RNA genes (tRNAs brown, rRNAs purple; other RNAs grey), GC content, GC skew.

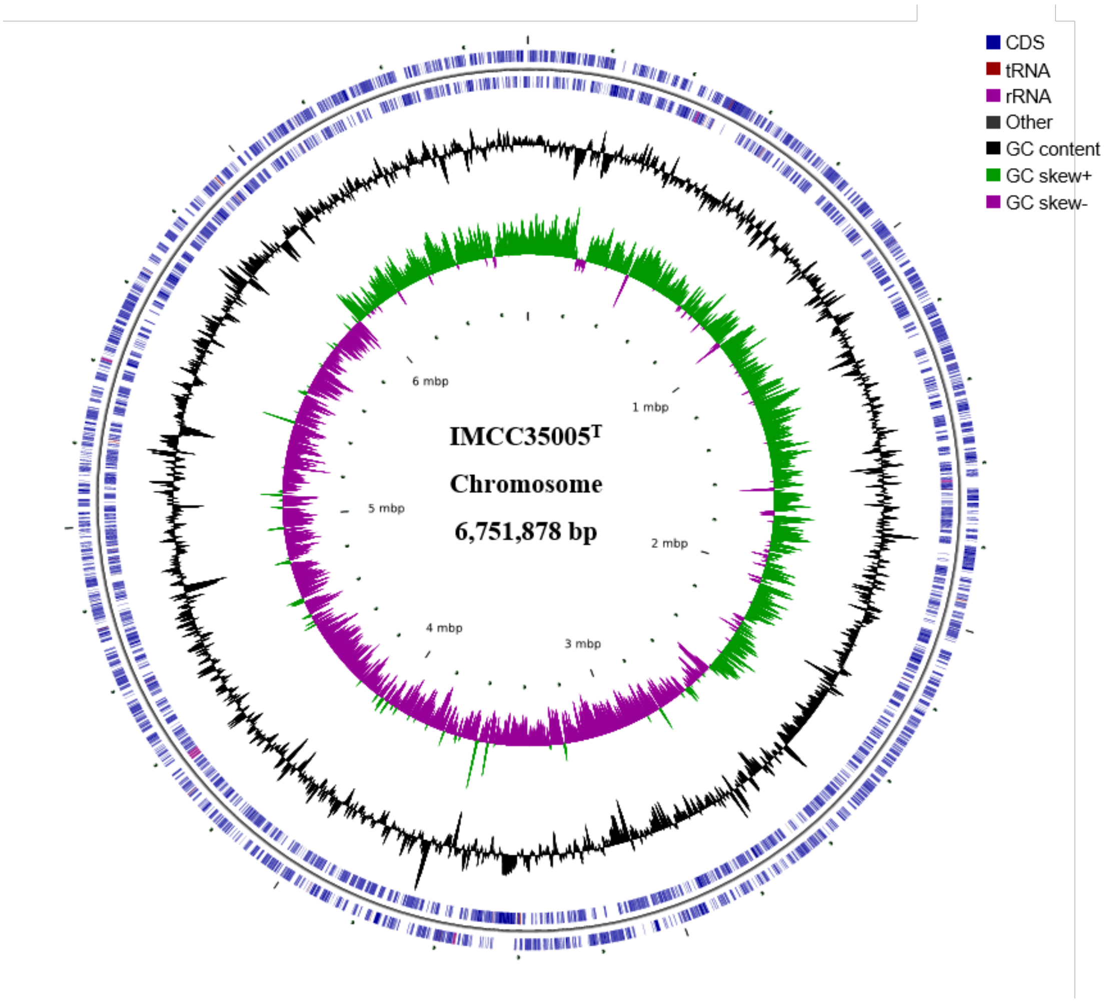

Fig. S3. Transmission electron micrographs of strains IMCC35004^T (a) and IMCC35005^T (b). Bar, 0.5 µm (a) and 1 µm (b).

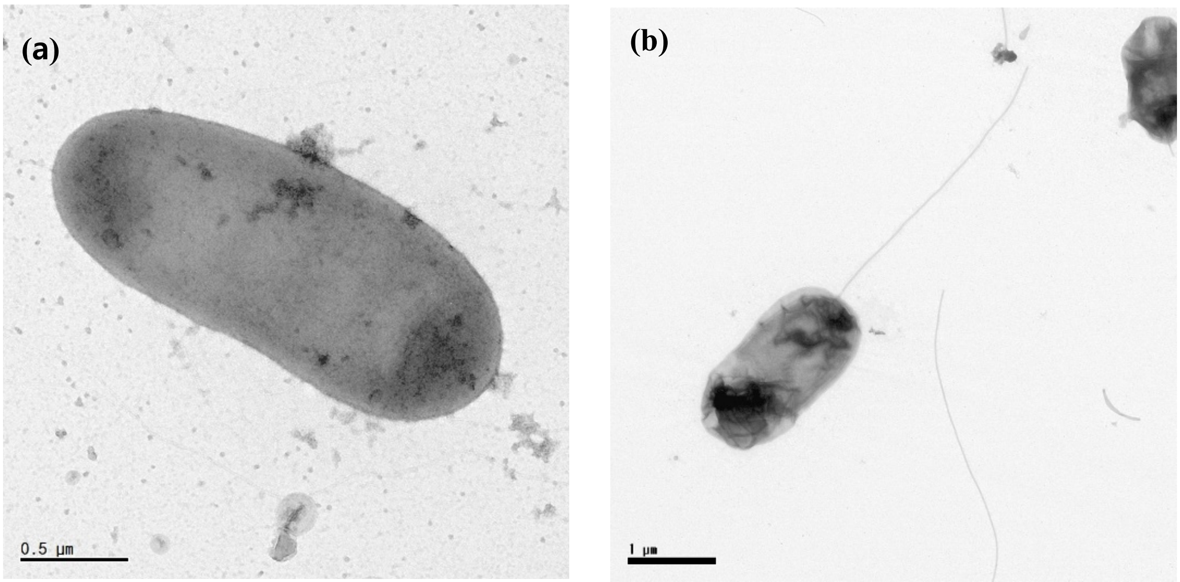

Fig. S4. Two-dimensional thin layer chromatography of polar lipids of strains IMCC35004^T (a), IMCC35005^T (b), *Desulfopila aestuarii* DSM 18488^T (c), and *Desulfotalea psychrophila* DSM 12343^T (d). PE, phosphatidylethanolamine; PG, phosphatidylglycerol; DPG, diphosphatidylglycerol; PL1-4, unidentified phospholipids; APL1-2, unidentified aminophospholipids; AL1-3, unidentified aminolipids; L1-8, unidentified lipids.

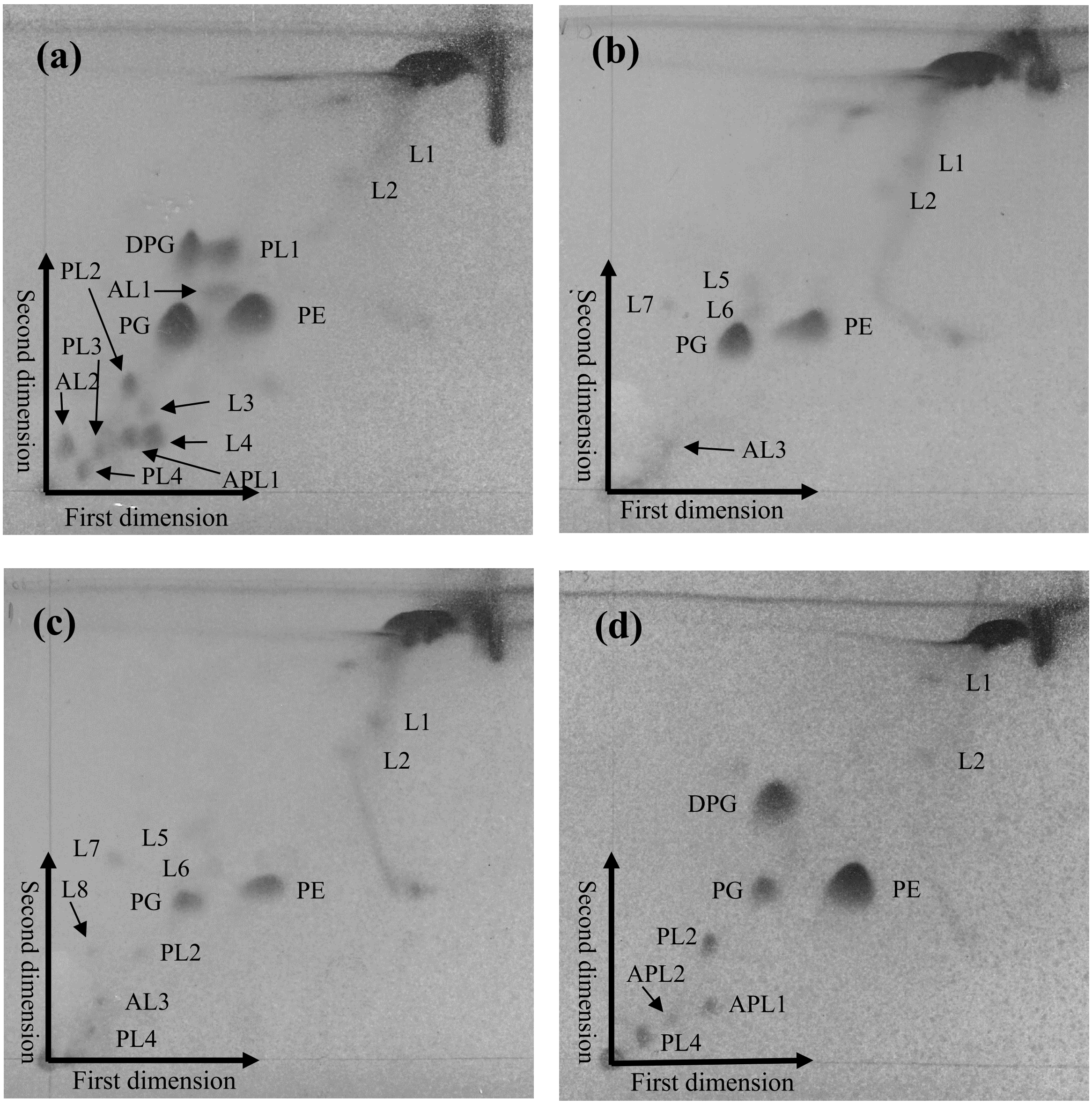

Figure S5. Venn diagram showing the orthogroups in the genomes of strains IMCC35004^T, IMCC35005^T, and closely related type strains of the family *Desulfocapsaceae*.

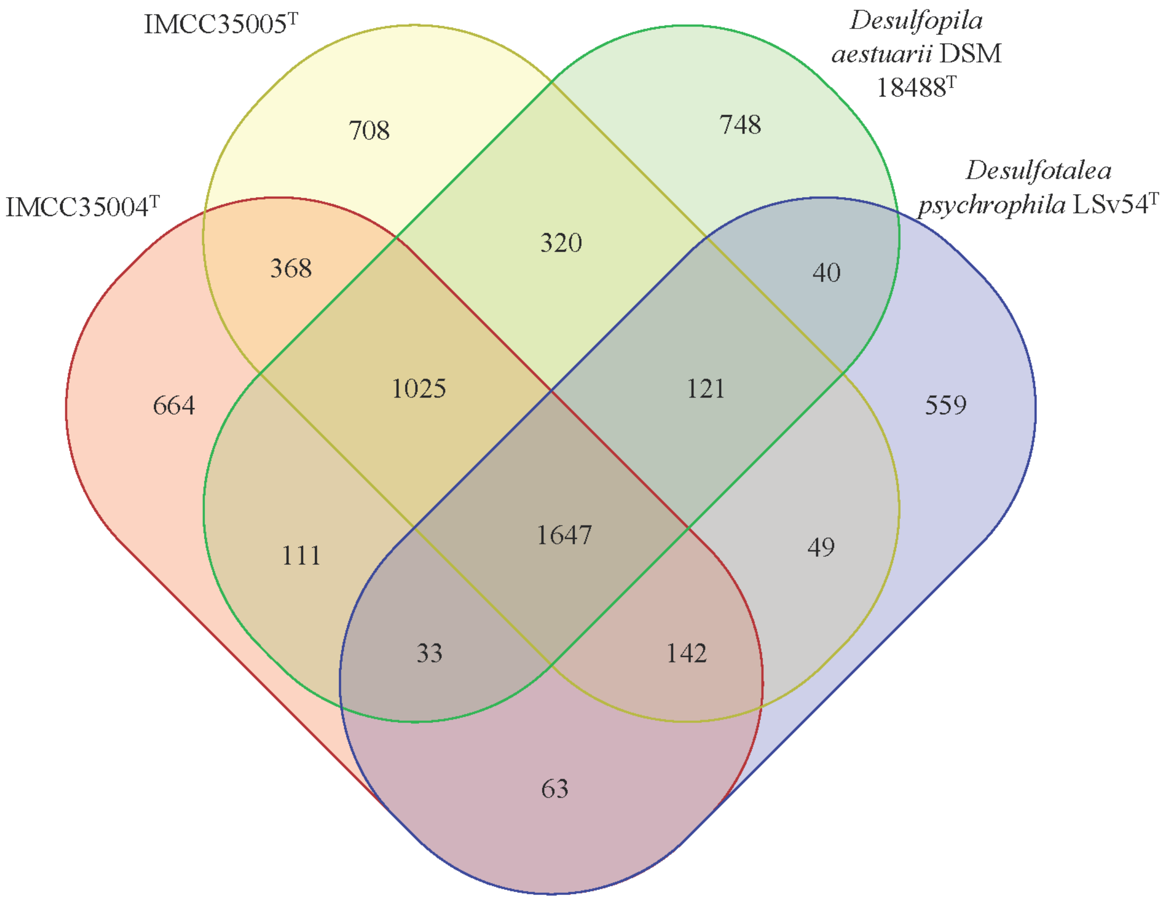

Supplement: Supplementary file 1 — Supplementary Information. [file 41598_2021_99469_MOESM1_ESM.pdf]
